# SignS: a parallelized, open-source, freely available, web-based tool for gene selection and molecular signatures for survival and censored data

**DOI:** 10.1186/1471-2105-9-30

**Published:** 2008-01-21

**Authors:** Ramon Diaz-Uriarte

**Affiliations:** 1 Statistical Computing Team, Structural Biology and Biocomputing Programme, Spanish National Cancer Center (CNIO), Melchor Fernández Almagro 3, Madrid, 28029, Spain

## Abstract

**Background:**

Censored data are increasingly common in many microarray studies that attempt to relate gene expression to patient survival. Several new methods have been proposed in the last two years. Most of these methods, however, are not available to biomedical researchers, leading to many re-implementations from scratch of ad-hoc, and suboptimal, approaches with survival data.

**Results:**

We have developed SignS (Signatures for Survival data), an open-source, freely-available, web-based tool and R package for gene selection, building molecular signatures, and prediction with survival data. SignS implements four methods which, according to existing reviews, perform well and, by being of a very different nature, offer complementary approaches. We use parallel computing via MPI, leading to large decreases in user waiting time. Cross-validation is used to asses predictive performance and stability of solutions, the latter an issue of increasing concern given that there are often several solutions with similar predictive performance. Biological interpretation of results is enhanced because genes and signatures in models can be sent to other freely-available on-line tools for examination of PubMed references, GO terms, and KEGG and Reactome pathways of selected genes.

**Conclusion:**

SignS is the first web-based tool for survival analysis of expression data, and one of the very few with biomedical researchers as target users. SignS is also one of the few bioinformatics web-based applications to extensively use parallelization, including fault tolerance and crash recovery. Because of its combination of methods implemented, usage of parallel computing, code availability, and links to additional data bases, SignS is a unique tool, and will be of immediate relevance to biomedical researchers, biostatisticians and bioinformaticians.

## Background

Many microarray studies involve human samples for which survival data are available. In the last two years there has been an increase in the number of new methods proposed for this kind of data [1-11]. Many of these papers have emphasized not only gene selection and survival prediction, but also "signature finding": discovering sets of correlated genes that are relevant for survival prediction. For end-users (e.g., biomedical researchers with microarray data for a sample of patients for which survival is known), however, most of these methods are not easily accessible, which might explain why many papers in the primary biomedical literature implement from scratch varied ad-hoc approaches in the context of survival prediction.

Unfortunately, in many cases, survival data are reduced to arbitrarily determined classes (such as dead or alive at a given, arbitrary, time), with the consequent loss of information, simply because tools for class prediction are much more widely available. Thus, tools for end users are badly needed that, while retaining user-friendliness, do not compromise statistical rigor.

Statistically, and in addition to appropriate analysis of censored data, such a tool should ensure that selection biases [12-15] are accounted for, to prevent overoptimistic assessments of the quality of the final model selected. Moreover, such a tool should also present the user with assessments of the stability of the results obtained: variable selection with microarray data (in general, in scenarios where the number of variables ≫ than the number of samples) can lead to many solutions that have similar prediction errors, but that share few common genes [16-18]. Choosing one set of genes without awareness of the multiple solutions can create a false perception that the selected set is distinct from the rest of the genes. Besides the statistical features, interpretation of results is enhanced if the tool provides additional information about "the interesting genes" such as PubMed references, Gene Ontology terms, and links to the UCSC and Ensembl databases and KEGG and Reactome pathways.

Such a tool should also try to incorporate the increasing availability of multicore processors and clusters made with off-the-shelf components. Since CPU performance has improved less than 20% per year since 2002 [19], the major opportunities for significant speed gains and the ability to analyze ever larger data sets with more complex analysis methods do not lie in faster CPUs. Rather, it is widely acknowledged that scaling to larger data sets and reducing user waiting time depends crucially on our ability to efficiently use parallel, distributed, and concurrent programming because of the increase in the available number of CPUs and CPU cores [20-23]. This trend affects even the laptop market (many laptops currently incorporate dual-core CPUs) and, therefore, the gains from parallel computing can be realized not only on computing clusters, but also in workstations and laptops.

Parallelization, such as provided by MPI [24], allows us to distribute the computations over a computing cluster, thus decreasing execution time. For an end user, parallelization can result in dramatic decreases in the time she/he needs to wait for the analysis to complete (see Benchmarking section). For developers, bioinformaticians, and biostatisticians, parallelization results in speed increases that ease method comparisons using extensive simulations and provides an example for the parallelization of further algorithms.

Regarding the user interface, web-based applications have been gaining popularity in bioinformatics among other reasons because they allow the development of user-friendly applications that do not require software installation or upgrades from the user [25]. In addition, web-based applications, if run in a computing cluster and implemented appropriately, make it possible to exploit parallelization.

Finally, source code availability under an open-source license allows researchers to improve upon the method, fix bugs, and verify claims by method developers, encourages reproducible research, and ensures that tool ownership resides in the international research community. These are all issues of particular concern in bioinformatics, where expedite progress builds upon previous research [26,27]. Moreover, the value of code availability is further enhanced when standard best practices in software development (see review and references in [28]) and the usual open source development mode [29], are followed.

We have developed a web-based tool, SignS (Signatures for Survival data), to fulfill the above needs. We know of no equivalent tool, and only BRB-ArrayTools [30], by R. Simon and A. P. Lam, provide somewhat similar functionality, but it is not web-based, does not ease accessing additional information, does not use parallel computing, and source code is not available. Thus, SignS is a unique tool, of immediate utility for biomedical researchers studying gene expression and its relation to survival (as is common, for example, in many cancer studies), and of broad appeal also to computational biologists, biostatisticians, and bioinformaticians because of the methods it implements and the combination of parallelization with web-based computing in an open-source application.

## Implementation

SignS is as a web-based application (and underlying R package) that provides four methods for gene selection with survival data: the method by Gui and Li [2], the approach of Dave et al. [1], a method that uses random forests with conditional inference trees [3,31], and a boosting method with component-wise univariate Cox models [3,32]. There are few methods that explicitly attempt to perform gene selection with survival data while preserving the identity of the individual genes and allowing the recovery of highly correlated genes. Moreover, there are few comparisons among the available methods, except those from [2,8,33-37]. In this context, we chose to implement these four very different approaches. The available comparisons indicate that penalization methods, specially those based on the *L*_1 _penalty, such as Lasso and LARS, tend to perform well and return results with relatively few genes, thus enhancing interpretation [33,35,36]. The method of Gui and Li can approximate the Lasso or LARS estimates, while selecting more relevant genes [2]. On the other hand, relatively heuristic and simple approaches such as those based on clustering and the idea of signatures can sometimes perform remarkably well, compared to sophisticated penalization approaches [36]. The method of Dave et al. [1] is one such method that attempts to explicitly return signatures for survival data. Finally, ensemble approaches are currently gaining popularity. The recent review by [34] has found that random forest-based methods, as in [31], can yield the best survival time predictions and, thus, we have also included this method. An alternative approach to using ensembles is via boosting, as in [3,32,38]; this approach has the advantage of providing for explicit variable selection and being computationally efficient, and has been shown to be competitive for at least some microarray data sets [3]. We have parallelized all the algorithms, providing significant decreases in user wall time (see below).

### Algorithms

Briefly, the steps of the method by Dave et al. [1] are: A) genes are filtered by p-value using a gene-by-gene Cox model. B) The retained genes are divided into two groups, those with a positive coefficient in the Cox model and those with a negative coefficient, and a hierarchical cluster is built for each of these two groups separately. C) A potential signature is a group of genes (a cluster) such that the minimal correlation between any two genes in the signature is above a (user-selected) threshold, and such that this cluster has between a minimum and a maximum number of genes (again, parameters set by the user). D) The numeric value of a component, signature in the sense of [1], is the average expression level of all the genes in a given component (i.e., for each sample, we compute the value of a signature as the average value, for that sample, of all the genes in that signature). E) Finally, we carry out variable selection using as starting point the best two-signature model; the variables used are the signature values computed in step D) above. Variable selection is carried out on the signatures, not on individual genes, and no gene can belong to multiple signatures. The main advantages of the approach of [1] are that it is easy to understand, that it explicitly attempts to return sets of correlated genes (signatures), and that the user is both forced to be explicit about, and allowed to choose, parameters with relatively straightforward interpretation (such as the minimum correlation of genes within a signature, or the maximum and minimum number of genes in a signature). Thus, the method of [1] is ideal for exploratory analysis, which is further enhanced by our additions, in particular the assessment of stability of solutions and functional annotation via IDconverter and PaLS (see "Results" section).

In contrast, the approach of Gui and Li [2] has two parameters but they rarely need to be tuned, and can instead be chosen by cross-validation. The complete method, including the dimensional reduction and the ability to capture correlated genes, follows from the penalization used (penalization is a general approach–with other examples being the Lasso and ridge regression– used to obtain estimates of the regression coefficients in high-dimensional spaces such as these, with few subjects and thousands of features). The solutions of this method depend on two parameters: a threshold parameter *τ *and an infinitesimal increment Δ*υ*. The threshold parameter *τ*, constrained between 0 and 1, is related to the amount of penalization, and larger values lead to a larger numbers of coefficients in the Cox model being set to 0 (i.e., to the selection of a smaller number of genes). The infinitesimal increment Δ*υ *affects the rate of change of the coefficients at each iteration of the algorithm. Note that, operationally, we can instead choose a sufficiently small Δ*υ*, and modify the number of iterations. [2] use cross-validation to choose the optimal parameters: a set of *τ *is decided in advance, and cross-validation is used to select the Δ*υ *(or, alternatively, the number of iterations), that minimizes the cross-validated partial likelihood (CVPL). The CVPL is a function of the difference in the log Cox's partial likelihoods for the models with and without the *i*th group of subjects. Once the optimal parameters are decided, it is immediate to obtain the fitted coefficients for the genes and the scores for subjects not in the sample. Thus, if we choose a sufficiently small Δ*υ*, no parameters need to be chosen by the user, as these are selected via cross-validation.

The approach of Hothorn et al. [3,31] for using random forests utilizes conditional inference trees as base learners. A conditional inference tree is built by recursively splitting the data into two groups according to the value of the covariables. First, we test the global null hypothesis of independence between any of the variables and the response. If this hypothesis is rejected, we select the variable with strongest association with the response, and implement a binary split in the selected input variable. We continue recursively (in each branch of the tree) until the global null is no longer rejected. The tests are carried out in a conditional inference framework (for details see [31]). A forest is built by fitting a conditional inference tree to each of the many (in our case, 500) bootstrap samples of the data. The type of response returned by these forests are Kaplan-Meir curves. In contrast to the previous two methods, there is no variable (gene) selection performed here although, implicitly, the construction of each tree involves choosing the best variable for a split. Following [34], by default we fit the forests to the best 200 genes, as determined from gene-wise Cox models, but the actual number of genes used is a parameter that can be chosen by the user of the application.

The last method included in SignS has been developed by Hothorn and colleagues [3,38] and uses boosting to analyze survival data when the number of variables is much larger than the number of samples. Boosting is a general iterative optimization method that, at each iteration, fits the best predictor variable (to reduce the appropriate loss function) and updates the estimates. The algorithm used, *L*_2 _Boosting, is equivalent to refitting residuals multiple times [32]. For survival data, [3,38] use component-wise univariate Cox models. Boosting requires choosing the number of boosting iterations, but as shown in [32], we can use cross-validation to select this parameter. This method performs variable selection, similar to the Lasso and other *L*_1_-penalty methods [32]. The genes selected are those with non-zero coefficients at the optimal number of boosting iterations.

### Implementation and parallelization

The method of [1] is available in both the web-based application and the underlying R code. The method of [2], as originally proposed, is only available in the R code because it is too slow for routine use in the web-based application; following a suggestion by J. Gui, the web application provides an implementation where only one threshold is used (see below). The core statistical functionality of both methods is written in R [39]. Where possible, computations have been parallelized using MPI (its LAM [Local Area Multicomputer]/MPI implementation) via the R-packages Rmpi [40], by H. Yu, and papply [41] by D. Currie. For the web-based application, the CGI (Common Gateway Interface), initial data validation, the setting-up and closing of the parallel infrastructure (booting and halting the LAM/MPI universes), and the fault-tolerance and crash recovery mechanisms, are written in Python.

The implementation of the approach in [1] follows closely the description of their method in the supplementary material to their paper. Our main departures from their implementation are: a) we do not split the data into two halves, but instead use cross-validation to assess model selection; b) it is unclear how the initial two-signature model was selected by the authors, and we choose the one with the largest likelihood, which in this case would be identical to using AIC, Akaike Information Criterion, as a criterion, since all two-signature models have the same number of parameters; c) it seems, from the supplementary material, that the authors used p-values in their forward variable selection, whereas we use AIC, generally a preferred criterion for model selection [42] (i.e., at each step we re-evaluate if any variables removed in previous steps should be incorporated, or any variables previously introduced into the model should be eliminated, using AIC as the criterion).

For this algorithm, we have parallelized computations over cross-validation runs, after experimenting with alternative parallelization schemes. Parallelizing the initial computation of gene-by-gene Cox models leads to decreases in speed because, in our setup, the communication costs are larger than the decrease in computing time; for instance, with a data set of 60000 genes and 160 arrays, the non-parallelized algorithm takes about 49 seconds in contrast to the 78 seconds of a parallelized algorithm that distributes the computational load evenly over 62 CPUs. Parallelization of the next step, clustering genes with positive and negative coefficients independently, could be split into two within each run, but the final step (stepwise model selection via AIC) is inherently sequential. Thus, we can minimize communication costs and simplify further modifications of the algorithm if the algorithm is parallelized at the cross-validation level. With this scheme, the total user wall time is the sum of the computing time of two runs of the algorithm –a first run with all the data, and another run corresponding to one of the cross-validations– plus the time invested in communication and data handling. In contrast, the user wall time of the purely sequential algorithm would be the sum of 11 runs of the algorithm –one for all data, and one for each of the cross-validation runs. For the method of [2] our code is based on the original code by the authors, with many modifications for speed improvement and parallelization. First, several common operations in the code were implemented using faster (sequential) code (e.g., using "crossprod" instead of the naive **X***'y*, vectorizing loops, rewriting expressions to use fewer steps, etc). Next, the code was parallelized. Taking into account the number of nodes we had available and the number of nodes that can often be used in off-the-shelf computing clusters, we parallelized the computations that search for the best *τ*. Following [2], we explore the six possible *τ *values 0, 0.2, 0.4, 0.6, 0.8, 1.0 and select the one that minimizes the CVPL, using 10-fold crossvalidation. Thus, we can parallelize the finding of the best *t *into 60 independent computations (10 searches at each of the candidate *τ*). Notice that parallelizing at this level yields increased speed even if no global/double/full cross-validation is performed. The speed-ups achieved with the code changes are discussed below. Because of speed issues with this method, the web-based application does not explore a range of *τ *values, but instead uses a single one, chosen by the user. J. Gui made us notice that, since their approach can only include genes, not delete them, it can result on small *τ *thresholds leading to the selection of many genes which can be false positives, and he suggested using only one or a few large *t *thresholds, and skipping cross-validation over the entire *τ *range, if time is at a premium (as in the web-based application). By default, therefore, the web-based application uses a *t *threshold of 0.9.

### Adding other algorithms: random forests and boosting

The above two methods have been implemented either almost from scratch, or after extensive modifications of the original code, including careful tuning of how to conduct the parallelization. To show how SignS can be extended with other algorithms, I have included the additional two methods in a much simpler way that also provide examples on how further methods can be incorporated. In both cases, there are R packages that implement each method, so essentially all we need to do is write several wrapper functions that call the necessary pre-existing functions, and that provide output in a way that can be used by SignS.

First, I have written some convenience functions that make it easier to produce formatted HTML with the results from the fits, and to obtain figures ("print.selected.genes", "print.cv.results", "writeForPaLS", "print.validation.results", "kmplots", "kmplots.validation"). These convenience functions will be used with the two new methods, and could be used directly, or with very minor modifications, if other algorithms are implemented.

Next, we will use the pre-existing code. The random forests method is available from the R package "party" [31], and can be downloaded from the R repositories. Likewise, the boosting method is available from the "mboost" R package [43]. For random forests, all we need to do is to wrap-up the existing function ("cforest") together with the other needed elements for SignS to operate. Following [34] we will perform a preliminary gene selection step, and after the model is fitted we will need to obtain predictions for each subject, to assess predictive performance (see below). Gene selection is carried out with a straightforward function, very similar to the one used in the method by Dave et al. to select genes. Subject prediction (either for cross-validation or with validation data) is based on the expected survival time, as explained in p. 109 and ff. of [44]; the mean survival time can be computed (although it is a biased estimate) even if survival at the largest observed time is much larger than 0.5. These three parts of the algorithm (gene selection –function "geneSelect"–, random forest –the original "cforest" function in package "party"–, prediction –"cf.mean.survtime") are wrapped in a single function ("my.cforest").

Next, we write another wrapper that will call this function repeatedly for cross-validation ("my.cforest.cv"). It is in the cross-validation where the algorithm is parallelized, so the 10 calls to cforest, one for each cross-validation fold, are run concurrently. The parallelization is straightforward since we are using the "papply" function (from the R package "papply"; see above).

Similar steps are followed for the boosting method. We define a function ("my.glmboost") that carries out all the needed steps: it calls the "glmboost" function in the mboost package for the initial boosting fit, and then the "cvrisk" function to select the best number of boosting iterations. The selected genes are the ones with non-zero coefficients at that best number of boosting iterations, and subject predictions (glmboost's linear predictors) are obtained using the model with that best number of boosting iterations. As above, we also write another wrapper function ("my.glmboost.cv") that will be used for cross-validating the predictive capacity of the approach and that is parallelized using "papply".

All the above code is available in the "SignS2.R" file (from the repositories, under SignS2/R) and is called from the "f1.R" file. The "f1.R" file is the actual R script, whereas SignS2.R is the underlying R package (by building a simple package, we not only make R namespace usage and management cleaner, but also ease the task of loading the code in multiple nodes when using parallelization). Finally, for the web-based application to operate, the Python code needs to be modified so that the user can select the newly implemented method and pass the appropriate parameters (only the number of genes to select for random forest).

### Crash recovery and fault tolerance

In distributed applications, partial failure (e.g., failure of one or more of the computing nodes) is unavoidable [45-47]. In our installation, we follow several complementary strategies to provide fault tolerance and crash recovery.

Shared storage space that uses RAID 50 provides protection against hard-disk failure, as well as access to results and data from nodes different from the one where computations started. Redundancy and load-balancing of the web-service is achieved with Linux Virtual Server with heartbeat and mon [48]. This setup ensures redundancy if one of the master nodes fails, and monitors the server nodes so that no HTTP requests are sent to non-responding nodes.

In addition to problems in the web-service and hard-disks, three sources of partial failure that can affect an ongoing calculation are network problems (which upset the LAM/MPI universe), MPI (or Rmpi) errors, and a crash in one of the nodes that are running a slave MPI job. All of these are recoverable errors and, therefore, there is no need to stop the complete calculation (forcing the user to relaunch the process) or halt indefinitely. Instead, SignS provides mechanisms to continue an ongoing calculation in case of the above sources of failure. First, the web-based application periodically examines MPI and Rmpi logs and existing LAM/MPI daemons to determine if any of the above problems have occurred. If a problem is found, a new LAM/MPI universe is booted. Before booting the new LAM/MPI universe, a script determines which nodes are currently alive and can run MPI processes and, if necessary, modifies the LAM/MPI configuration files accordingly. Once the new LAM/MPI universe is successfully created, a new R process is launched. The R code includes checkpoints so that calculations are not started again from the beginning but only continued from the point they were stopped.

Note that errors in our R code, since they are not recoverable, are functionally equivalent to completion of the calculations. The application monitorizes R logs and currently running R processes and, if any errors are detected, the calculation is aborted immediately, a message returned to the user, and the problem logged to allow for prompt fixing.

To ensure that the application is working properly a test is launched every hour from a machine external to the cluster. The test is one from the functional testing suite (see above) and verifies both the user interface and the parallelization infrastructure. If there are any errors, an email is immediately sent to the author and other system administrators.

## Results

### Functionality

SignS provides estimates of the performance of the final model using 10-fold CV (cross-validation). To assess predictive performance we use a simple and common [1,2,6-8,10,11,33,49-51] strategy: splitting the test samples into several (2, 3, or 4) groups based on their predicted scores (or predicted survival for random forests), and comparing the survival functions of these groups. It must be emphasized that the predicted scores are obtained from a full (or double: [15]) cross-validation, so the predicted scores for a sample correspond to the CV-fold for which that sample never participated in any of the steps leading to the final model.

If a validation data set is provided, the performance of the final model is also evaluated against this validation data set. The validation data set is only used to assess predictive performance, and is not used in any way to build the model.

To assess the stability of the results obtained, we report the number of signatures and the identity of the genes in each signature for the run with the original sample and the 10 CV runs, as well as tables with number (and percentages) of common genes in different runs. The list of these signatures and genes can be sent to our application PaLS [52] to examine PubMed references, Gene Ontology terms, KEGG pathways or Reactome pathways that are common to a user-selected percentage of genes and/or signatures. In other words, the shared features of each signature or set of selected genes can be examined with respect to Gene Ontology terms, KEGG and Reactome pathways, and PubMed references. Tables with output from each run include clickable links to our application IDClight [53] which provides additional information, including mapping between gene and protein identifiers, PubMed references, Gene Ontology terms, and KEGG and Reactome pathways.

SignS can run in platforms that range from a laptop to a cluster of workstations. Our installation runs on a cluster of 30 computing nodes, each with two dual-core AMD Opteron CPUs and 6 GB of RAM. In our implementation, additional nodes provide load-balancing, high-availability, and shared storage. We also incorporate a careful scheme for fault tolerance and crash recovery (see section "Crash recovery and fault tolerance"). The input for the web-based application are either plain text files, or files that come from other tools of the Asterias suite [54]. Further documentation and examples for the web-based application are available from its on-line help [55]. SignS has been running in production use for over a year and a half; monthly users in the last seven months, from 1 May to 30 November of 2007, are 490 (May), 490 (June), 270 (July), 390 (August), 680 (September), 780 (October), and 800 (November). Bug-tracking is available from [56]. SignS also includes a test suite that uses FunkLoad [57]; the tests allow to verify that the user interface and numerical output are working, thus ensuring appropriate quality control and regression testing.

The web-based application is accessible from [58]. All source code, including the web-based application, R code, and functional tests, are available from Launchpad [59] and [60] under open source licenses, GNU GPL for the R package (required for compatibility with the R and BioConductor packages used) and the Affero Public license for the rest of the code.

### Benchmarking

**Figure 1:**
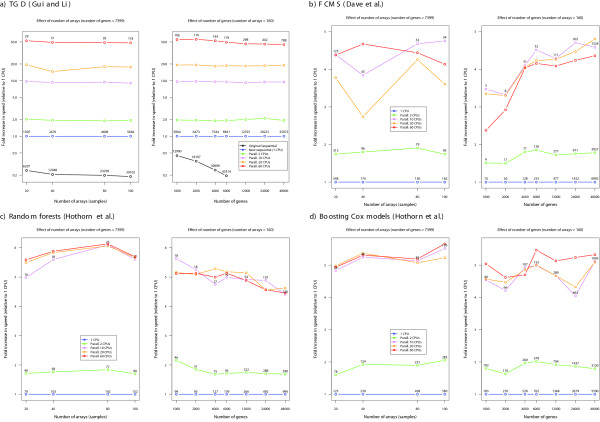
**Fold increase in speed (ratio of user wall times) of R code from code changes to sequential code (in a) and parallelization (a and b)**. a) Timings from functions "gdcvpl" (original code) and its equivalent "tauBestP" (SignS), which use cross-validation to find the best parameters. b, c, d) Timings using analysis that include cross-validation of the final model. Numbers on top of points: user wall times in seconds. Benchmarks obtained in an otherwise idle cluster with 30 nodes, each with two dual-core AMD Opteron 2.2 GHz CPUs and 6 GB RAM, running Debian GNU/Linux and a stock 2.6.8 kernel, version 7.1.2 of LAM/MPI and version 2.1.4 (patched) of R. DLBCL data set from [4]; when number of arrays, *n*, ≤ 160 and number of genes, *p*, ≤ 7399, we use the first *n *arrays and the first *p *genes of the data set. For number of genes *p > *7399 we expand the data set creating new genes from the previous (real) ones with Gaussian noise added.

The speedups achieved in the method by [2] with our code changes (see "Implementation and parallelization") and parallelization are shown in Figure 1a), using realistic ranges of numbers of genes and samples (arrays). Before any parallelization, rewriting the sequential code leads to speed improvements of factors between 2 (= 1/0.5) and 5 (= 1/0.2). These speed improvements are larger as we increase the number of arrays and, specially, the number of genes. Parallelization leads to further, and large, increases in speed, which are almost linear with the number of slave processes (concurrently running R processes). With 60 slave processes, there is a speed improvement of a factor of about 50: in parallel computing [24,61] other factors in addition to number of CPUs can become limiting, in our case most likely bandwith and latency of inter-node communication, and potential bottlenecks from memory and cache in nodes made of dual-core processors [22]. Moreover, the rewritten code (either on a single CPU or parallelized) shows good scalability: running time increases sublinearly with both number of arrays and number of genes (e.g., doubling the number of genes results in an increase in computing time which is less than double).

**Figure 2:**
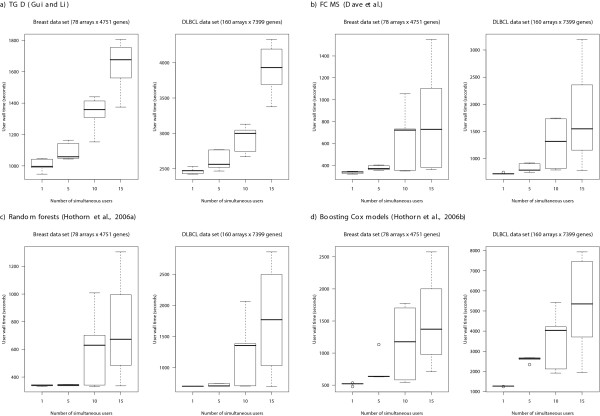
**User wall time of the web-based application**. User wall time as a function of number of simultaneous users for two different (and real) data sets, obtained from [4]. To increase the realism of simultaneous accesses, there is delay of 5 seconds between simultaneous accesses, as might be expected, for example, from a classroom demonstration (i.e., when simulating 10 simultaneous users, the cluster is actually receiving new connections over a 10 * 5 second period, with one new connection every 5 seconds). Shown are box-plots of user wall times from several runs: 5 runs for 1 and 5 users, 10 runs for 10 users and 15 runs for 15 users. Hardware and software the same as in Figure 1.

For the other three methods (FCMS, random forests, boosting) parallelization results in more modest gains (see Figure 1b). First, there is no gain in speed when we use more than 10 slave processes. This is what we would expect, since we parallelize over cross-validation runs (see "Implementation and parallelization" and "Adding other algorithms" for rationale) and adding further nodes can not result in increased speed since those are not used. In all three cases, however, the increases in speed with only 2 CPUs are almost equivalent to doubling execution speed. For FCMS, the scaling with number of genes is superlinear (e.g., doubling the number of genes results in increases in computing time which are more than double), a result of the superlinear scaling of clustering and model selection with number of genes; changes in computing time with number of arrays, however, do not show a consistent increase with number of arrays and, with small number of arrays, computing time can be much larger, because the model selection step takes much longer (the number of models to consider is often much larger with 20 arrays than with 80 or 100 arrays). For random forest, increases in number of genes only result in noticeable changes in computing time for large numbers of genes (6000 and over), which is to be expected since the random forest algorithm, itself, always uses at most 200 genes, so we will not notice the increase in computing with number of genes through random forest, but rather through the preliminary gene selection step (and possible communication costs). Increases in computing time with number of arrays, when using random forests, are modest and sublinear. With boosting, increases in computing time with number of genes are more noticeable, but only become linear over 6000 genes; increases in computing time with number of arrays are almost linear. Figure 2 shows the time a user will wait for the web-based application to complete (user wall time) as a function of the number of simultaneous users using the application in that very moment. (When using a slow internet connection these numbers will increase and, e.g., uploading a data set of 8.5 MB, such as DLBCL, to the application can take over 5 minutes). As can be seen from the figure, SignS can handle a large number of simultaneous users and shows excellent scalability with number of users. This is the result of both the parallelization of the computations and the load balancing of the non-parallelized code. Note that situations with 10 or more simultaneous users are completely unrealistic, since the average number of daily users of SignS is less than 30. The above benchmarks, though, show that SignS can handle even those high numbers of users, which makes it suitable for classroom use.

Scripts for all benchmarks are available from the repositories.

## Discussion

We have developed a web-based tool (and underlying R package), SignS, for gene selection and signature building from microarray data when we have censored and survival data. SignS presents several unique features that make it very relevant for both applied and methodological work.

First, there are no alternative web-based applications for these types of analysis. Source code and packages are available from some other approaches (e.g., [3,4,8,31]), but most of them are out of the direct reach of biomedical researchers, as they all require a minimal proficiency with R and BioConductor. There is only one alternative application with biomedical researchers as target users, BRB-ArrayTools [30]. In contrast to BRB-ArrayTools, SignS is available as a web-based application (BRB-ArrayTools is only available as an Excel add-on) and, therefore, SignS does not require any specific operating system or application, just a web browser. In addition, the complete code of SignS is available as open source (and we follow standard best practices in software development and the usual open source development mode). Moreover, SignS implements four very different, complementary methods of analysis. Finally, the availability of both the source code and the scripts for each run immediately provide for "reproducible research": the complete results can be reconstructed as the user has the code that implements the entire sequence of steps and the parameter settings used. "Reproducible research" is a problem of great importance with complex analysis sequences that is gaining attention in the analysis of genomic data [62]. By themselves, these features make SignS a unique and pioneering tool.

Second, SignS is one of the very few genomic analysis tools to use parallel computing. Parallel computing is crucial to allow further improvements in user wall time and to analyze ever larger data sets: betting on single CPU performance improvements is no longer reasonable, given both the slow increase in CPU speed in the last five years, and the increased availability of multi-core and multi-CPU computers, from laptops and workstations to clusters. Our results, using realistic scenarios regarding number of genes and samples, show that: a) our web-based implementation of SignS can handle a large number of simultaneous users with good scalability (see Figure 2); b) the performance improvements of parallelization can be harvested even in dual-core laptops and personal computers: relative speed increases of the R code with 2 CPUs are around 2× for TGD, and 1.8× for the other three methods (see Figure 1).

Moreover, by its usage of parallel computing, SignS sets a standard in terms of implementing tools that take advantage of recent advances in hardware and computer science. SignS represents a rare case example of combining a user-friendly web-based interface with parallel computing, –including fault-tolerance and crash recovery– that, by making the full source code available, allows other researchers to build upon our work and, by the usage of open source licenses, ensures that the code remains owned by the research community. Extending upon our work is further eased because we use no Python-specific web-frameworks nor R extensions as a web-based application; therefore, the logic of the application (including the web-based application and the fault tolerance mechanisms) could be programmed in any other language and the computational engine could be different from R.

Third, SignS strives to ease the biological interpretation of results using functional annotation of results via links to additional data bases that allow mapping between gene and protein identifiers, PubMed references, Gene Ontology terms, and KEGG and Reactome pathways. Moreover, SignS further enhances the critical assessment of results by allowing the examination of possible multiple equivalent solutions (using cross-validation and analysis of similarities among results of the different runs).

## Conclusion

SignS fills an important need as a user-friendly, web-based application for gene selection and signature finding with survival data. It is also a unique tool (by its combination of methods implemented, usage of parallel computing, code availability, and links to additional data bases), and thus it will be of immediate interest to biomedical researchers, biostatisticians and bioinformaticians. Moreover, SignS sets a high standard for future applications of this kind.

## Availability and requirements

**Project name: **SignS

**Project home page: **

**Operating system: **Platform independent (web-based application)

**Programming language: **R, Python

**Other requirements: **A web browser.

**License: **None for usage. Web-based code: Affero GPL (open source). R code: GPL (open source).

**Any restrictions to use by non-academics: **None.

## Abbreviations

CGI, Common Gateway Interface; GO, Gene Ontology; KEGG, Kyoto Encyclopedia of Genes and Genomes; LAM, Local Area Multicomputer; MPI, Message Passing Interface.

## Authors' contributions

RD-U carried out all the work described and wrote the manuscript.
